# Supplementary material for: Functional characterisation of rare variants in genes encoding the MAPK/ERK signalling pathway identified in long-lived Leiden Longevity Study participants
Source: GeroScience. 2025 May 29;48(1):625–45. doi: 10.1007/s11357-025-01699-2 (PMC12972501; doi:10.1007/s11357-025-01699-2)
Supplement: Supplementary file 1 — Supplementary file1 (PDF 953 KB) [file 11357_2025_1699_MOESM1_ESM.pdf]

Supplementary Information

Functional characterisation of rare variants in genes encoding the MAPK/ERK signalling pathway identified in long-lived Leiden Longevity Study participants

Baghdadi M., Hinterding H., et al.

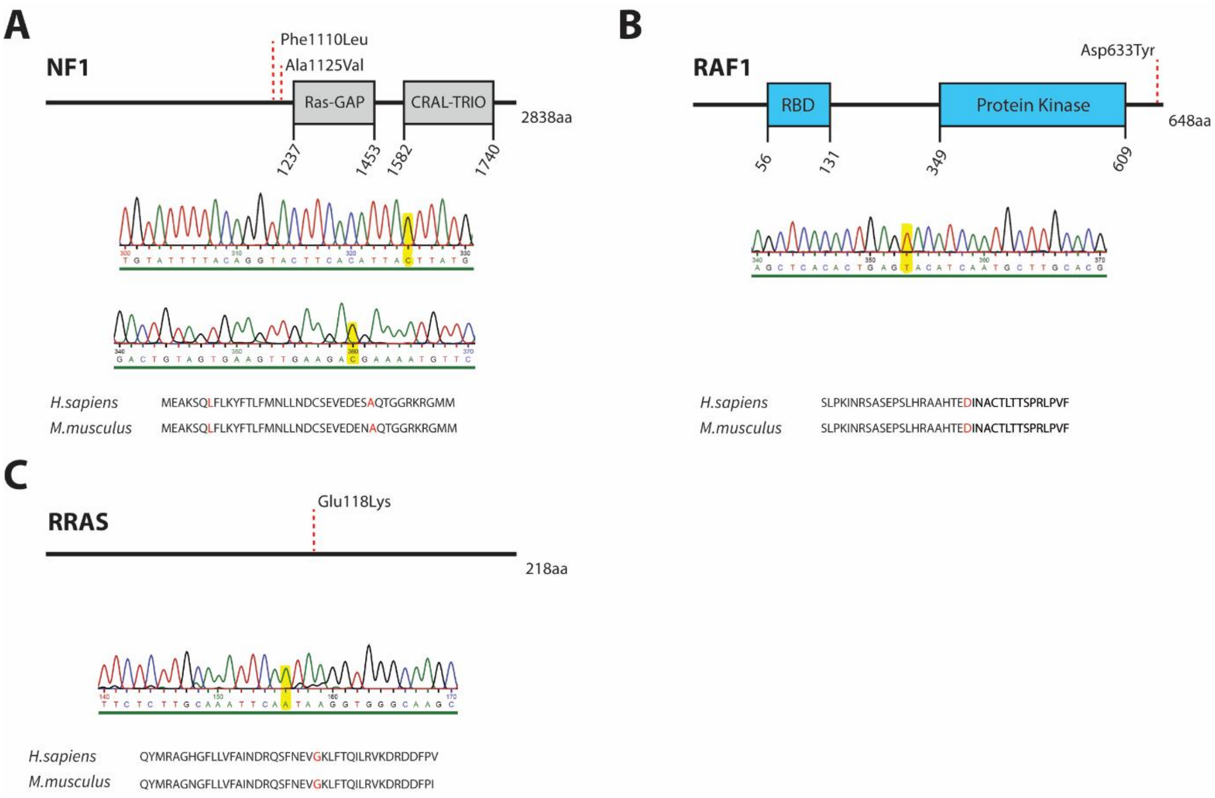

Figure S1 | Location and conservation of rare genetic variants in genes involved in the MAPK/ERK signalling pathway for which transgenic mESC lines were created.
(**A**-**C**) Schematic representation highlighting the features (based on www.uniprot.org) of the NF1 (**A**), RAF1 (**B**), and RRAS (**C**) proteins, including the location of the identified variants (denoted by dashed red lines), the DNA sequence resulting from Sanger sequencing validation (with the mutation highlighted in yellow), as well as the sequence homology between humans and mice. Ras-GAP; Ras-specific GTPase-Activating Protein, CRAL-TRIO; Cellular RetinALdehyde-binding protein-Trio, RBD; Ras-Binding Domain.

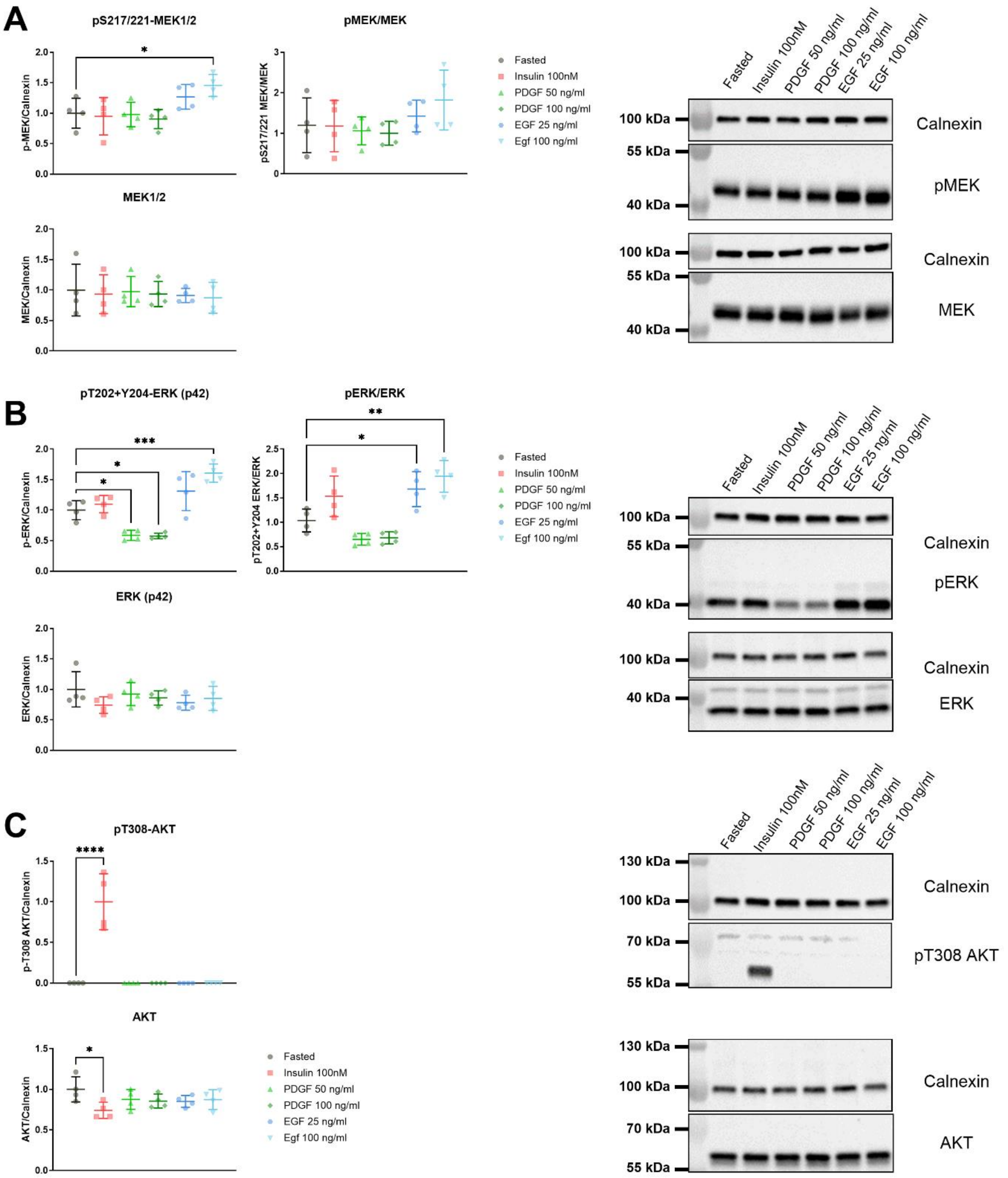

Figure S2 | MAPK/ERK and IIS pathway-related effects after insulin, PDGF, or EGF stimulation in wildtype mESCs.

(**A**) EGF stimulation (100 ng/ml for 2 min.) was sufficient to significantly up-regulate phosphorylated MEK1/2 (Ser 217/221) over total MEK1/2 after fasting (6 hr.) in wildtype mESCs. (**B**) EGF stimulation (25 ng/ml and 100 ng/ml for 2 min.) was sufficient to significantly up-regulate the ratio of phosphorylated (T202+Y204) ERK1/2 over total ERK1/2 after fasting (6 hr.) in wildtype mESCs. (**C**) Insulin stimulation (100 nM for 10 min.) was the only growth factor to significantly up-regulate the phosphorylation of AKT (T308) over total AKT. Error bars represent standard deviation. Data was analysed using a one-way ANOVA and Dunnett's post hoc test. $*P < 0.05$, $**P < 0.01$, $***P < 0.001$, $****P < 0.0001$.

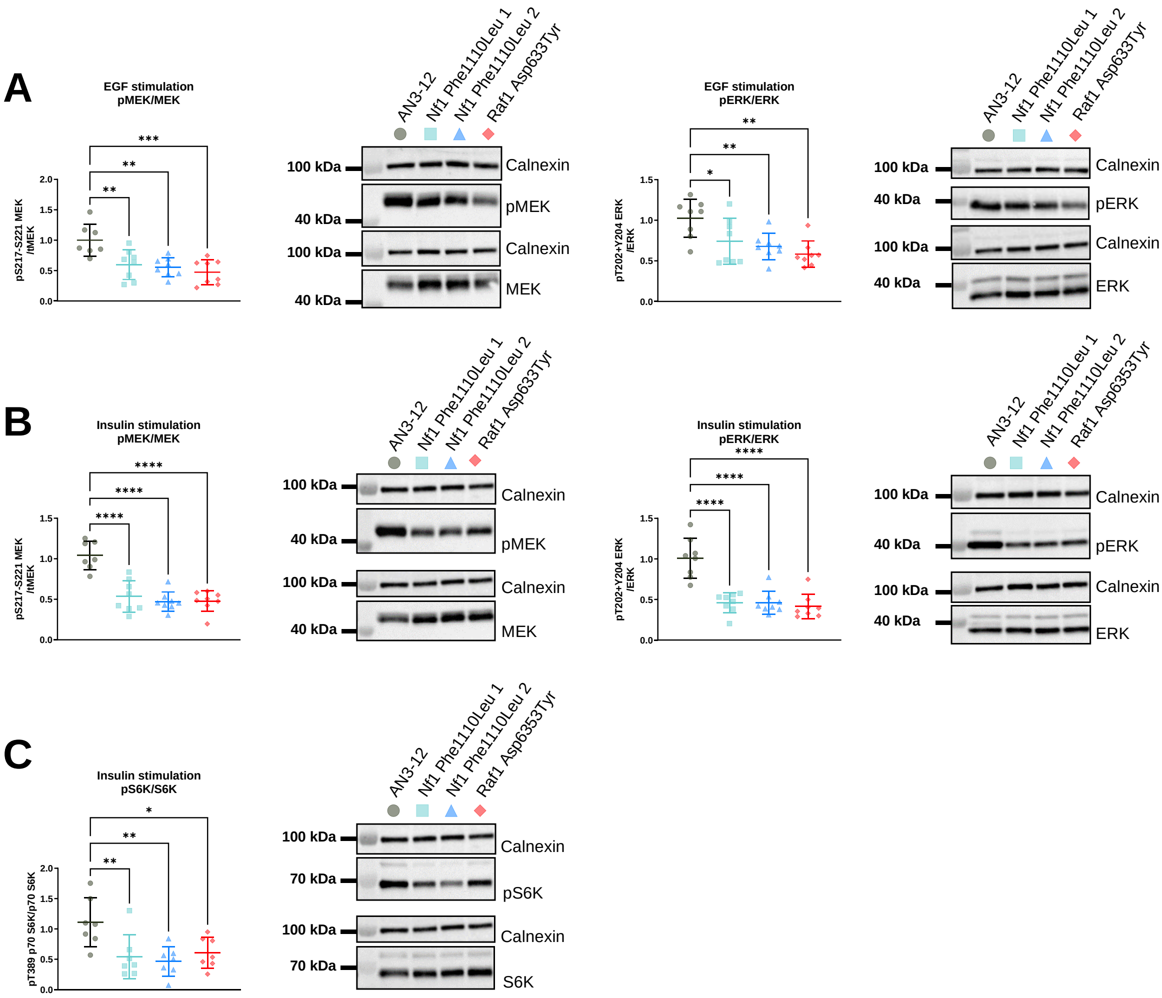

Figure S3 | MAPK/ERK and IIS pathway-related effects of the NF1^{Phe1112Leu} and RAF1^{Asp633Tyr} variant mESC lines after EGF and insulin stimulation.

(**A**) Significant reduction of phosphorylated MEK1/2 (Ser217/221) over total MEK1/2 and phosphorylated ERK1/2 (Thr202+Tyr204) over total ERK1/2 in the mESC lines harbouring the NF1^{Phe1112Leu} and RAF1^{Asp633Tyr} variants after EGF stimulation (100 ng/ml for 2 min.). (**B**) Significant reduction of phosphorylated MEK1/2 (Ser217/221) over total MEK1/2 and phosphorylated ERK1/2 (Thr202+Tyr204) over total ERK1/2 in the mESC lines harbouring the NF1^{Phe1112Leu} and RAF1^{Asp633Tyr} variants after insulin stimulation (100 nM for 10 min.). (**C**) Significant reduction of phosphorylated p70 S6K (Thr389) over total p70 S6K in the mESC lines harbouring the NF1^{Phe1112Leu} and RAF1^{Asp633Tyr} variants after insulin stimulation (100 nM for 10 min.). Error bars represent standard deviation. Data was analysed using a one-way ANOVA and Dunnett's post hoc test. *$P < 0.05$, **$P < 0.01$, ***$P < 0.001$, ****$P < 0.0001$.

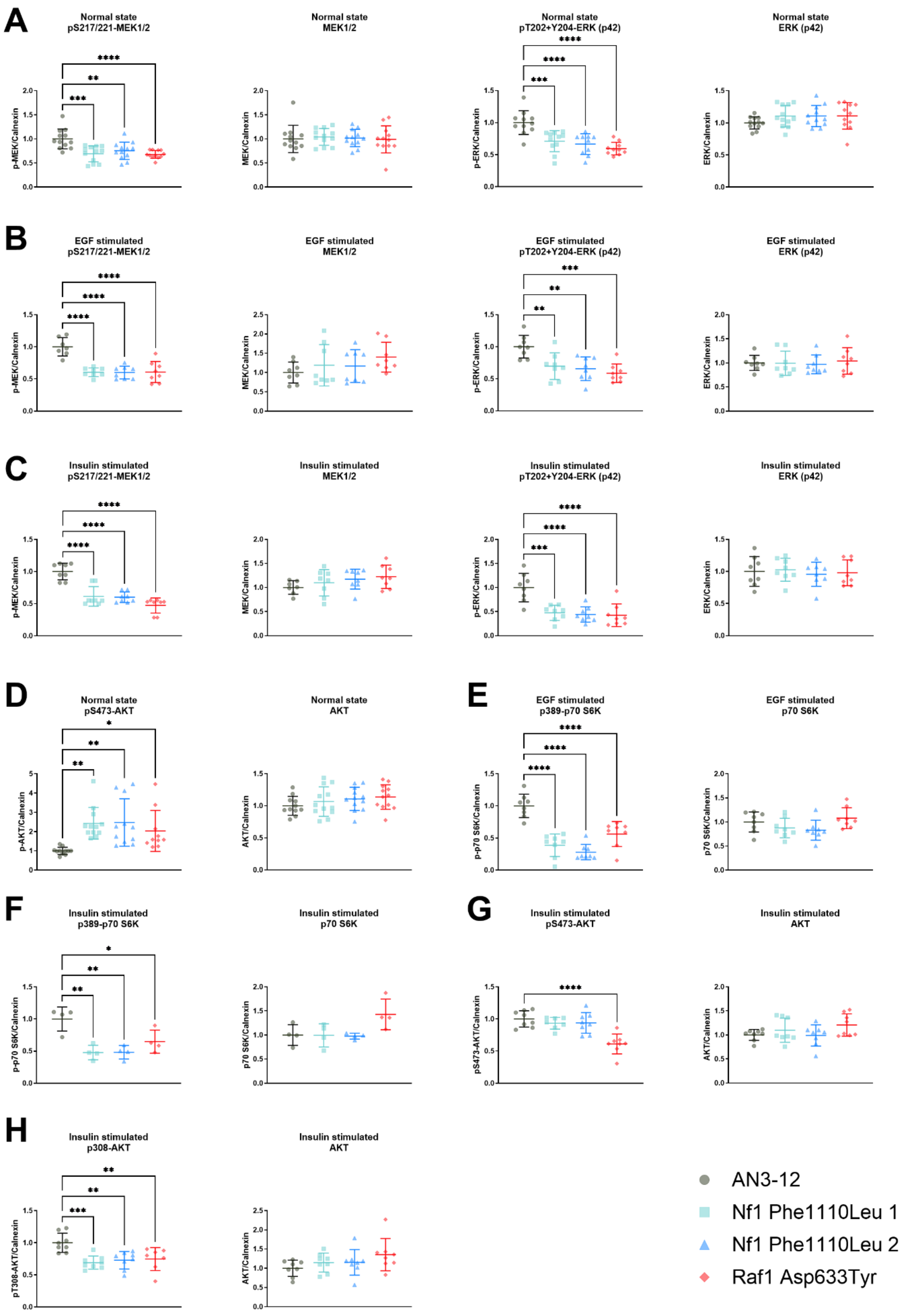

Figure S4 | Phosphorylated and total MAPK/ERK and IIS pathway-related protein levels in the NF1^{Phe1112Leu} and RAF1^{Asp633Tyr} variant mESC lines.

(**A-C**) Significant reduction of phosphorylated MEK1/2 (Ser217/221) and phosphorylated ERK1/2 (Thr202+Tyr204), but not total MEK1/2 and ERK1/2, in the mESC lines harbouring the NF1^{Phe1112Leu} and RAF1^{Asp633Tyr} variants maintained in normal growth medium (**A**), as well as after EGF stimulation (100 ng/ml for

2 min.) (**B**) and insulin stimulation (100 nM for 10 min.) (**C**). (**D**) Significant increase of phosphorylated AKT (Ser473), but not total AKT, in the mESC lines harbouring the $NF1^{Phe1112Leu}$ and $RAF1^{Asp633Tyr}$ variants maintained in normal growth medium. (**E-F**) Significant reduction of phosphorylated p70 S6K (Thr389), but not total p70 S6K, in the mESC lines harbouring the $NF1^{Phe1112Leu}$ and $RAF1^{Asp633Tyr}$ variants after EGF stimulation (100 ng/ml for 2 min.) (**E**) and insulin stimulation (100 nM for 10 min.) (**F**). (**G**) Significant reduction of phosphorylated AKT (Ser473), but not total AKT, in the mESC line harbouring the $RAF1^{Asp633Tyr}$ variant. (**H**) Significant reduction of phosphorylated of AKT (Thr308), but not total AKT, in the mESC lines harbouring the $NF1^{Phe1112Leu}$ and $RAF1^{Asp633Tyr}$ variants after insulin stimulation (100 nM for 10 min.). Error bars represent standard deviation. Data was analysed using a one-way ANOVA and Dunnett's post hoc test. *$P < 0.05$, **$P < 0.01$, ***$P < 0.001$, ****$P < 0.0001$.

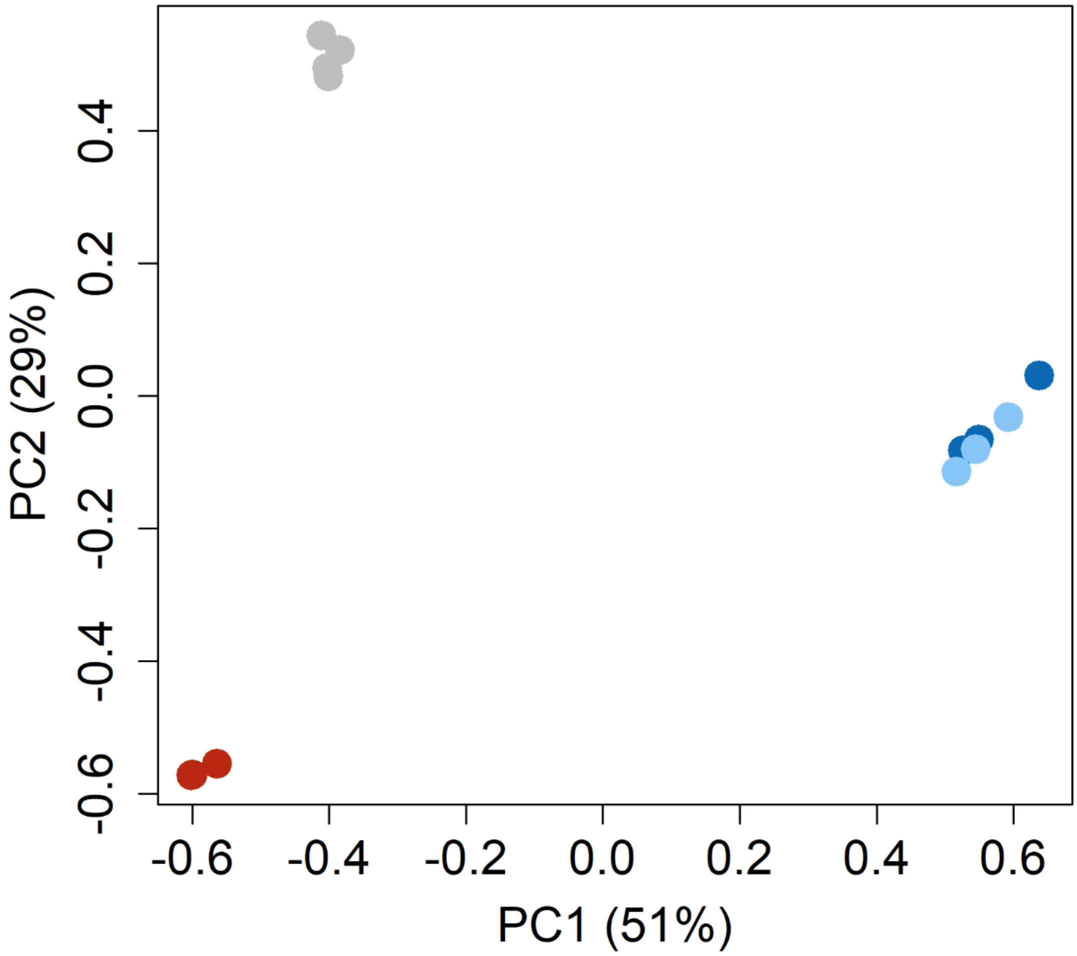

Figure S5 | Principal component analysis of proteomics data of the NF1^{Phe1112Leu} and RAF1^{Asp633Tyr} variant mESC lines.

TMM normalised clustering confirms the two independent cell lines harbouring the NF1^{Phe1112Leu} variant to be identical (light blue and dark blue dots). The RAF1^{Asp633Tyr} cell line (red dots) clusters differently from the wildtype (grey dots) and NF1^{Phe1112Leu} variant cell lines.

Table S1 | Overview of used guide RNAs.

Target mutation	Vector backbone	gRNA1	gRNA2
$NF1^{Ala1127Val}$	pSpCas9n(BB)-2A-GFP	GAAGATGAAAATGCACAAAC	CTTCACTACAGTCATTCAAA
$NF1^{Phe1112Leu}$	pSpCas9n(BB)-2A-GFP	GAAGATGAAAATGCACAAAC	CATAAATAATGTGAAGTACC
$RAF1^{Asp633Tyr}$	pSpCas9n(BB)-2A-GFP	GCACGCTGACTACATCCCCA	AGTGTGAGCTGCCCGATGCA
$RRAS^{Glu118Lys}$	pSpCas9n(BB)-2A-GFP	TCTTGCAGTTTCAATGAGGT	GCATGAGGCACCACCTTAGC

Table S2 | Overview of used single-stranded DNA oligonucleotides.

Target mutation	ssODN
NF1^{Ala1127Val}	TCTCTGTATTTTCCAGGTACTTCACATTATTTATGAATCTTTTGAATGACTGTAGTGAAGTTGAAGACGAAAATG**T**TCAAACCGGTGGCAGGAAACGTGGCATGTCTCGGAGGCTGGCAT
NF1^{Phe1112Leu}	CATGGTGTAAGGTCAGTCTCTGTATTTTACAGGTACTTCACATTA**C**TTATGAACCTTTTGAATGACTGTAGTGAAGTTGAAGACGAAAATGCTCAAACGGGTGGCAGGAAACGTGGCATG
RAF1^{Asp633Tyr}	AAAATCAACAGGAGCGCCTCTGAGCCTAGCCTGCATCGGGCAGCTCACACTGAG**T**ACATCAATGCTTGCACGCTGACTACATCCCCAAGACTACCAGTCTTCTAGCTGATGATGTAGCTG
RRAS^{Glu118Lys}	GGATGCTGGTGGGCCTGGCTGAGCACGGGGCAGGTTACTGCTAAGGTGGTACCTCATGCTACTGACTTCTCTTGCAAATTCAAT**A**AGGTGGGCAAGCTCTTCACACAGATCCTCAGAGTC

The target mutations are highlighted in red, silent PAM and restriction site mutations are highlighted in orange.

Table S3 | Overview of oligonucleotides used for genotyping.

Target gene	Forward Primer	Reverse Primer
Raf1	TTAGATCATCTTCATGGTAGGCC	ACAGGGCCTTAACTTCATATTGC
Nf1	GTTGAAATTTTCCTTGTAATGAATC	TCCTTCCTCCTGGTTAATGG
Rras	GCAAGAGGAATTTGGTGCCA	GCAGGCAGGCAAGAAGTAAG

Table S4 | Overview of primary antibodies used for western blotting.

Antigen	Source	Dilution	Vendor #ID
Phospho-p44/42 MAPK (Erk1/2) (Thr202+Tyr204)	Rabbit	1:1000	Cell Signaling #4370
p44/42 MAPK (Erk1/2) total	Rabbit	1:1000	Cell Signaling #4695
Phospho-Akt (Thr308)	Rabbit	1:1000	Cell Signaling #9275
Phospho-Akt (Ser473)	Rabbit	1:1000	Cell Signaling #4060
AKT total	Rabbit	1:1000	Cell Signaling #9272
Phospho-p70 S6 Kinase (Thr389)	Rabbit	1:1000	Cell Signaling #9205
p70 S6 Kinase total	Rabbit	1:1000	Cell Signaling #9202
Phospho-MEK1/2 (Ser217/221)	Rabbit	1:1000	Cell Signaling #9154
MEK1/2 total	Rabbit	1:1000	Cell Signaling #9122
Phospho-CHK1 (Ser345)	Mouse	1:1000	Santa Cruz Biotechnology #8408
CHK1 total	Rabbit	1:1000	Cell Signaling #2348
Phospho-gamma-H2AX (Ser139)	Rabbit	1:1000	Abcam #11175
gamma-H2AX total	Rabbit	1:1000	Cell Signaling #2577
Calnexin	Rabbit	1:1000	Merck Millipore #208880

Table S5 | Overview of TaqMan Assays used for qPCRs.

Gene	TaqMan Assay ID
Gapdh	Mm99999915_g1
c-Myc	Mm00487804_m1
Foxo3	Mm01185722_m1
Nfe2l2	Mm00477784_m1
Ets1	Mm01175819_m1
Ets2	Mm00468973_m1
Etv1	Mm00514804_m1
Etv4	Mm00476696_m1
Etv5	Mm00465816_m1
Etv6	Mm01261325_m1

Table S6 | Gene list MAPK/ERK signalling pathway.

Ensembl stable ID	Gene name
ENSG00000078061	*ARAF*
ENSG00000128272	*ATF4*
ENSG00000157764	*BRAF*
ENSG00000120129	*DUSP1*
ENSG00000143507	*DUSP10*
ENSG00000111266	*DUSP16*
ENSG00000158050	*DUSP2*
ENSG00000108861	*DUSP3*
ENSG00000120875	*DUSP4*
ENSG00000138166	*DUSP5*
ENSG00000139318	*DUSP6*
ENSG00000164086	*DUSP7*
ENSG00000184545	*DUSP8*
ENSG00000130829	*DUSP9*
ENSG00000138798	*EGF*
ENSG00000146648	*EGFR*
ENSG00000134954	*ETS1*
ENSG00000157557	*ETS2*
ENSG00000139083	*ETV6*
ENSG00000010030	*ETV7*
ENSG00000177885	*GRB2*
ENSG00000174775	*HRAS*
ENSG00000133703	*KRAS*
ENSG00000109270	*LAMTOR3*
ENSG00000169032	*MAP2K1*
ENSG00000126934	*MAP2K2*
ENSG00000100030	*MAPK1*
ENSG00000102882	*MAPK3*
ENSG00000079277	*MKNK1*
ENSG00000099875	*MKNK2*
ENSG00000172680	*MOS*
ENSG00000158186	*MRAS*
ENSG00000196712	*NF1*
ENSG00000213281	*NRAS*
ENSG00000072062	*PRKACA*
ENSG00000142875	*PRKACB*
ENSG00000165059	*PRKACG*
ENSG00000154229	*PRKCA*
ENSG00000166501	*PRKCB*
ENSG00000126583	*PRKCG*
ENSG00000110786	*PTPN5*
ENSG00000143851	*PTPN7*
ENSG00000153233	*PTPRR*
ENSG00000132155	*RAF1*
ENSG00000116473	*RAP1A*
ENSG00000127314	*RAP1B*
ENSG00000109756	*RAPGEF2*
ENSG00000145715	*RASA1*
ENSG00000155903	*RASA2*
ENSG00000058335	*RASGRF1*
ENSG00000113319	*RASGRF2*
ENSG00000172575	*RASGRP1*
ENSG00000068831	*RASGRP2*
ENSG00000152689	*RASGRP3*
ENSG00000171777	*RASGRP4*
ENSG00000117676	*RPS6KA1*
ENSG00000071242	*RPS6KA2*
ENSG00000177189	*RPS6KA3*
ENSG00000162302	*RPS6KA4*
ENSG00000072133	*RPS6KA6*

**Ensembl stable ID**	**Gene name**
ENSG00000126458	*RRAS*
ENSG00000262489	*RRAS2*
ENSG00000160691	*SHC1*
ENSG00000129946	*SHC2*
ENSG00000148082	*SHC3*
ENSG00000185634	*SHC4*
ENSG00000115904	*SOS1*
ENSG00000100485	*SOS2*

Table S7 | Results of the overrepresentation analysis using KEGG and MSigDB hallmark gene sets of the RAF1^{Asp633Tyr} and NF1^{Phe1112Leu} variant mESC lines.

Comparison	ID	Description	GeneRatio	BgRatio	p-value	FDR-adjusted p-value
RAF1^{Asp633Tyr} Down	mmu01100	Metabolic pathways	294/1083	735/3366	2.45×10^{-7}	7.92×10^{-5}
	mmu00982	Drug metabolism - cytochrome P450	10/1083	11/3366	9.02×10^{-5}	0.015
	mmu05323	Rheumatoid arthritis	13/1083	17/3366	2.21×10^{-4}	0.024
	mmu04966	Collecting duct acid secretion	11/1083	14/3366	4.77×10^{-4}	0.039
	mmu00480	Glutathione metabolism	19/1083	31/3366	7.65×10^{-4}	0.049
		HALLMARK_MTORC1_SIGNALING	71/718	154/2209	1.83×10^{-4}	0.009
		HALLMARK_GLYCOLYSIS	55/718	119/2209	9.32×10^{-4}	0.022
RAF1^{Asp633Tyr} Up	mmu04512	ECM-receptor interaction	20/842	29/3366	7.09×10^{-7}	2.18×10^{-4}
	mmu03010	Ribosome	52/842	117/3366	2.47×10^{-6}	3.81×10^{-4}
	mmu04514	Cell adhesion molecules	18/842	28/3366	1.19×10^{-5}	0.001
	mmu05412	Arrhythmogenic right ventricular cardiomyopathy	19/842	32/3366	3.50×10^{-5}	0.002
		HALLMARK_MITOTIC_SPINDLE	62/577	168/2209	8.88×10^{-4}	0.043
NF1^{Phe1112Leu} Down	mmu05323	Rheumatoid arthritis	14/863	17/3366	1.49×10^{-6}	4.77×10^{-4}
		HALLMARK_INTERFERON_GAMMA_RESPONSE	32/600	69/2209	4.01×10^{-4}	0.019
NF1^{Phe1112Leu} Up	mmu05412	Arrhythmogenic right ventricular cardiomyopathy	20/915	32/3366	2.80×10^{-5}	0.009
	mmu03010	Ribosome	51/915	117/3366	7.45×10^{-5}	0.012
	mmu03008	Ribosome biogenesis in eukaryotes	33/915	69/3366	1.73×10^{-4}	0.018
		HALLMARK_MTORC1_SIGNALING	68/649	154/2209	3.84×10^{-5}	0.001
		HALLMARK_MYC_TARGETS_V2	29/649	52/2209	5.22×10^{-5}	0.001

GeneRatio; size of the overlap of genes of interest with that node / size of the overlap of geneset with all members of the collection of geneset, BgRatio; genes of interest in the gene set / unique genes in the background genes.
